# Toward the Rational Design of More Efficient Mo_2_C Catalysts for Hydrodeoxygenation–Mechanism and Descriptor Identification

**DOI:** 10.1021/acscatal.3c03728

**Published:** 2023-10-05

**Authors:** Raghavendra Meena, Johannes Hendrik Bitter, Han Zuilhof, Guanna Li

**Affiliations:** † Biobased Chemistry and Technology, Wageningen University, Bornse Weilanden 9, 6708 WG Wageningen, The Netherlands; ‡ Laboratory of Organic Chemistry, Wageningen University, Stippeneng 4, 6708 WE Wageningen, The Netherlands; § School of Pharmaceutical Sciences and Technology, Tianjin University, 92 Weijin Road, Tianjin 300072, People’s Republic of China

**Keywords:** molybdenum carbide, hydrodeoxygenation, heteroatom doping, linear-scaling relationships, descriptors

## Abstract

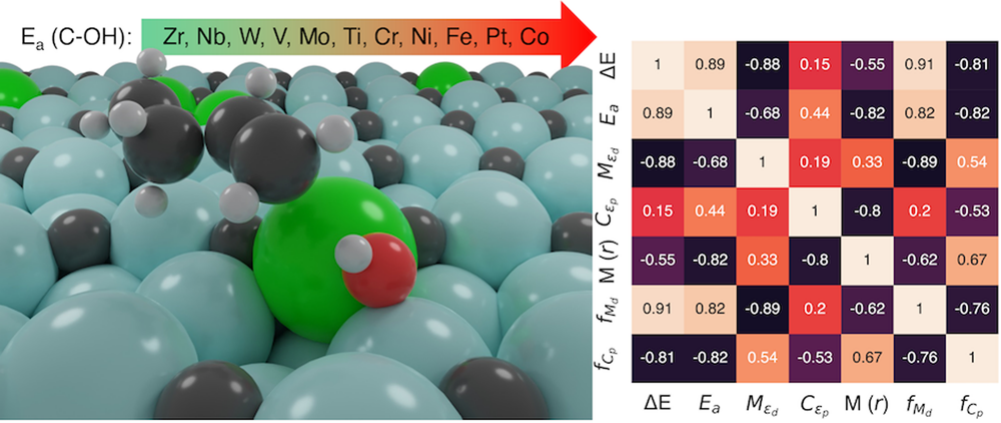

Viable alternatives to scarce and expensive noble-metal-based catalysts are transition-metal carbides such as Mo and W carbides. It has been shown that these are active and selective catalysts in the hydrodeoxygenation of renewable lipid-based feedstocks. However, the reaction mechanism and the structure–activity relationship of these transition-metal carbides have not yet been fully clarified. In this work, the reaction mechanism of butyric acid hydrodeoxygenation (HDO) over molybdenum carbide (Mo_2_C) has been studied comprehensively by means of density functional theory coupled with microkinetic modeling. We identified the rate-determining step to be butanol dissociation: C_4_H_9_*OH + * → C_4_H_9_* + *OH. Then we further explored the possibility to facilitate this step upon heteroatom doping and found that Zr- and Nb-doped Mo_2_C are the most promising catalysts with enhanced HDO catalytic activity. Linear-scaling relationships were established between the electronic and geometrical descriptors of the dopants and the catalytic performance of various doped Mo_2_C catalysts. It was demonstrated that descriptors such as dopants’ d-band filling and *atomic radius* play key roles in governing the catalytic activity. This fundamental understanding delivers practical strategies for the rational design of Mo_2_C-based transition-metal carbide catalysts with improved HDO performance.

## 1 Introduction

The catalysis community is facing two grand challenges: (1) tackling over-reliance on nonrenewable fossil-based resources for materials, chemicals, and fuel production; (2) design and discovery of efficient catalysts based on earth-abundant elements. These two challenges need to be addressed together.^1−3^ The use of biomass as feedstock together with abundant transition-metal carbide based catalysts is therefore an integrated approach that can address both of these challenges.^4^

Traditionally, platinum-group metals (PGMs) are well-known for their high catalytic activity and stability while catalyzing a wide range of reactions involved in biomass valorization.^5−8^ However, PGMs are scarce, hence expensive, and susceptible to deactivation by impurities such as sulfur-based compounds (SO_*x*_) and nitrogen-based compounds (NO_*x*_) present in the feedstocks.^9−11^ Therefore, much work has been done to find viable alternative catalysts to PGMs, of which transition-metal carbides (TMCs),^12−18^ especially molybdenum carbide (Mo_2_C)^19−25^ and tungsten carbide (W_2_C),^23−26^ are most promising because they exhibit PGM-like properties due to their comparable electronic structures.

TMCs are synthesized by introducing C atoms in the transition metal’s lattice. Electronic structure studies showed that the valence s-p electrons from the C atoms combine with the s-p-d electrons from the transition metal, providing the TMCs with catalytic properties similar to those of PGMs.^14,16,27−30^ Also, it has been reported that the activity of a bulk transition-metal catalyst is largely governed by the presence of either a high or, in contrast, a specific low density of d electrons close to the Fermi level (*E*_F_).^27,28^ In the case of PGMs, the *E*_F_ is present in a region where a high density of d-electrons is present, while the *E*_F_ lies in the region of a low density of d electrons in the case of bulk TMs. However, in the case of TMCs, *E*_F_ lies in the region of the intermediate level of d-electron states. Thus, the electronic structure of TMCs resembles that of PGMs, and as a result TMCs gain PGM-like catalytic properties. Furthermore, TMCs show resistance to deactivation by impurities in the bulk^11,24^ and are also a popular choice because of their mechanical hardness, thermal stability, and conductivity.^12^

Experimentally, TMCs have been shown to be active for reactions such as hydrogenation, hydrodeoxygenation, isomerization, methanation, etc.^19−25,31^ For instance, Oyama et al.^14^ reported that in reforming *n*-hexane at 670 K, a tungsten carbide (β-W_2_C) catalyst shows yields close to that of Pt/SiO_2_ (8.6% vs 11.6%), and β-W_2_C exhibits even better selectivity for isomerization products compared to that of Pt/SiO_2_ (71% vs 60%) for the same reaction. Stellwagen et al.^23^ performed experiments determining the activity and selectivity differences in W_2_C and Mo_2_C supported on carbon nanofibers for a stearic acid hydrodeoxygenation reaction. They observed that W_2_C catalysts were selective for alkene products, while Mo_2_C catalysts were selective toward oxygenates. The obtained alkenes and oxygenates are platform chemicals for synthesizing a wide range of value-added products. Interestingly, in contrast to W_2_C and Mo_2_C, conversion of stearic acid using PGMs such as Pd and Pt catalysts primarily yields heptadecane via a decarboxylation pathway, which shows that TMCs favor different mechanisms.^32^

Although the catalytic performance of TMCs has been investigated by experiments, an understanding of the underlying mechanisms and the intrinsic nature of active sites in many of the aforementioned reactions is still quite challenging. Density functional theory (DFT) has been used to identify the nature of active sites at the atomic level. For example, Shi et al. studied the structure and stability of different facets of the orthorhombic Mo_2_C using DFT. These authors took into consideration different facets such as (001), (010), (110), (100), (110), (101), (011), and (111) with all possible surface terminations (Mo; C; Mo/C) and found that the (011) facet with the mixed termination (Mo/C) is the most stable surface, followed by the (101) facet with mixed termination (Mo/C).^33^ Also, Wang et al. studied the CO adsorption equilibrium over (001), (101), and (201) facets of hexagonal Mo_2_C catalyst using DFT. These authors provided phase diagrams showing that a stable CO coverage can be obtained by tuning the temperature (*T*) and partial pressure (*p*) of CO for different facets of Mo_2_C.^34^ Additionally, these authors showed that the stability of a particular Mo_2_C facet can be tuned as a function of carbon chemical potential and the differences in surface termination (Mo vs Mo/C) explicitly influence the CO activation mechanism.^35^ Furthermore, DFT has been used to study reaction mechanisms over different Mo_2_C catalysts: e.g., Ren et al. showed that Mo_2_C is a highly selective catalyst for conversion of biomass-derived oxygenate to unsaturated hydrocarbons as it selectively cleaves C–O bonds and not C–C bonds.^21^ Shi et al.^36^ studied the HDO reaction mechanism of butyric acid to butane over a hexagonal Mo_2_C (101) surface within the DFT framework. The stepwise reaction mechanism was provided, and butanol dissociation was identified as the rate-determining step (RDS). These authors also studied water formation on Mo_2_C (001) and Mo_2_C (101) facets and established that the barrier for water formation is sensitive to the choice of the active Mo_2_C facet. Oliveira et al.^37^ also studied the HDO reaction mechanism of acrylic acid over orthorhombic Mo_2_C (001) within the DFT framework. Their proposed mechanism led to the conclusion that an alkane and water should be the main products, in line with experimental observations by Sousa et al.^38^ Another conclusion was that TMCs predominantly attack the C=O bonds as shown by Ren et al.^21^ as well, while PGMs favor activating C=C scission. Moreover, Wang et al. explored the change in adsorption energies of CO, H_2_, H_2_O, and CO_2_ over a stable hexagonal Mo_2_C (101) catalyst upon doping with metals such as Fe, Co, Ni, Cu, Pt, and Pd. These authors observed a strong influence of loading (0% vs 25% vs 50%) on the adsorption energies due to change in the electronic structure upon doping, which results in strong electron transfer from the metal to the surface in the case of transition metals, while this effect is less prominent in the case of Pt and Pd.^39^ These computational studies provided a fundamental basis for exploring the intrinsic nature of other promising Mo_2_C-based catalysts for the HDO reaction and established that butyric acid is a good model for investigating the HDO reaction mechanisms.

On the other hand, even though the catalytic activity and reaction mechanism of Mo_2_C catalysts are known to some degree from previous experimental and theoretical investigations, it is still not clear what the structure–activity relationships are. Many strategies have been proposed in the literature to understand such relationships, and most of them are based on the Sabatier principle^40^ and Brønsted–Evans–Polanyi (BEP)^41,42^ principle.^43−45^ For instance, Michaelides et al.^46^ performed *ab initio* calculations to determine a linear scaling relationship between the activation energies and reaction enthalpies for a broad range of surface-catalyzed reactions. Nørskov et al. have implemented the Sabatier and BEP principles in their works^47−51^ and developed a model, i.e., the Hammer–Nørskov model,^52^ to demonstrate that trends in adsorption energies over transition metals are governed by interactions between an adsorbate’s valences states and a metal’s d states. More recently, Gong et al.^53−55^ have used similar approaches in their studies for screening catalysts by means of interpretable intrinsic descriptors of activity. For example, these authors used a multidimensional approach in studying the redox activity of vanadium oxides upon doping.^53^ They showed that the p-band center is the most crucial descriptor governing the activity, and other descriptors emerging from the coordination environment such as an unoccupied d-band center and s- and d-band fillings also play important roles in tuning the oxygen activity.Such structure–activity relationships shed light on the intrinsic laws governing the external catalytic activities and provide an efficient approach for catalysts screening.

In the current study, we investigated the mechanism of all reaction steps involved in the hydrodeoxygenation (HDO) of the butyric acid reaction over the orthorhombic Mo_2_C (101) surface by DFT, to provide a detailed insight into the formation of oxygenates, alkenes, and alkanes. Even though the HDO mechanism of butyric acid has previously been studied over hexagonal Mo_2_C (101) catalyst,^36^ it was interesting to see how the most stable orthorhombic Mo_2_C catalyst promotes the HDO reaction. Hence, we used a stable corrugated orthorhombic Mo_2_C (101) catalyst.^56^ Throughout the text we refer to the orthorhombic phase of Mo_2_C as β-Mo_2_C. Butyric acid has been used as a model compound to mimic the nature of long-chain fatty acids derived from the biobased feedstock.^36^ The results obtained using DFT were then used as an input for a microkinetic model to derive surface coverages and degree of rate control (DRC) coefficients. DRC analysis showed that butanol dissociation is the RDS to which the overall rate of the reaction is the most sensitive. Subsequently, we doped the Mo site involved in this RDS with a range of transition metals to tune the activity of β-Mo_2_C. In addition, the key descriptors governing the structure–activity relationships of the doped β-Mo_2_C catalysts were identified. By analyzing the structure–activity relationships, we identified that dopants’ d-band filling and *atomic radius* are the two most relevant descriptors correlating well with the activity of doped β-Mo_2_C catalysts.

## 2 Computational Details

All the DFT^57,58^ calculations were performed with the Vienna *ab initio* Simulation Package (VASP.5.4.4 and VASP.6.2.1).^59,60^ The generalized gradient approximation (GGA) with PBE exchange and correlation functional was used to account for the exchange-correlation energy.^61^ The *electron–ion* interactions were described using the projected augmented wave (PAW) method and the plane-wave (PW) basis set.^59,60^ The convergence criterion for energy calculation and structure relaxation was set to an SCF threshold of 10^–5^ eV and a maximum force threshold of 0.05 eV/Å. Γ-centered *k*-meshes with sizes of 6 × 6 × 6 and 2 × 2 × 1 were used for sampling the Brillouin zone in the case of bulk and slab models, respectively. Gaussian-type smearing with a width of 0.05 eV was applied for the electronic energy density of states. To identify the transition states, the climbing-image nudged elastic band (CI-NEB) method^62,63^ was used, and frequency analysis was performed to confirm that there was only one imaginary frequency along the reaction coordinate. Dipole corrections were applied in the vacuum (*z*) direction. For CI-NEB calculations, a maximum force threshold of 0.10 eV/Å was implemented. The vdW interactions were described by the DFT-D3BJ method developed by Grimme et al.^64,65^

To study the catalytic activity of Mo_2_C toward HDO of butyric acid, we choose the orthorhombic (β) phase of Mo_2_C. From experimental phase diagrams, β-Mo_2_C has been concluded to be the most stable phase of Mo_2_C.^33,66^ X-ray diffraction (XRD) and DFT studies have also confirmed that β-Mo_2_C is the most stable phase.^67−69^ Low Miller index (100, 001) surfaces of β-Mo_2_C have been used for theoretical studies because of their high activities for hydrogenation reactions; however, they are less likely to dominate the surface area of as-synthesized β-Mo_2_C catalysts as predicted by Tacey et al.^36,37,56^ Therefore, in this work, we use the (101) catalytic surface as it has a more corrugated structure, making it a more dynamic catalytic surface and fetching unique catalytic properties.^56^ Additionally, the reactivity is explicitly dependent on the species present on the surface. In the case of β-Mo_2_C (101), the surface can have either one of Mo and C species or it can have both Mo and C species *aka* mixed-termination. It has been reported that mixed termination (Mo–C–Mo) enhances the stability of the catalytic surface but, at the same time, also provides a ridged structure which explicitly influences the reactivity of the surface.^56^ Hence, we considered using a mixed-terminated β-Mo_2_C (101) catalytic surface in this study.

The bulk structure of β-Mo_2_C was fully relaxed, and we obtained the lattice parameters *a* = 4.75 Å, *b* = 5.23 Å, and *c* = 6.05 Å, which are in good agreement with the experimentally reported values (*a* = 4.74 Å, *b* = 5.21 Å, *c* = 6.03 Å).^67^ From the optimized bulk, we cleaved a (101) surface and built a unit cell with a thickness of nine atomic layers (three stoichiometric layers of Mo_2_C), as shown in Figures S1 and S2 in the Supporting Information. A vacuum distance of 15 Å in the *z* direction was introduced to minimize interaction with the periodic images. After relaxing the atoms in the unit cell, we created a supercell of size 2 × 3 × 1, which was deemed to be a big enough surface for the HDO reaction. The bottom six atomic layers of the supercell were fixed to reduce the computational cost of the calculations and mimic the bulk.

The adsorption energies (*E*_ads_), reaction energies (Δ*E*), and activation barriers (*E*_a_) were calculated as follows:
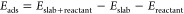
1
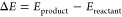
2
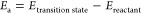
3

Here, *E*_slab__+reactant_ is the total energy of the slab with a reactant adsorbed on it, *E*_slab_ is the total energy of the clean slab, *E*_reactant_ and *E*_product_ are the total energies of the reactant and product of each elementary reaction step, respectively, and *E*_transition state_ is the total energy of the transition state (TS). The zero-point energies have been corrected for all of the adsorption and desorption steps.

For descriptor identification, Bader charge analysis was done using code written by Henkelman et al.^70^ and the electronic structure parameters such as M (d-band center), NM (p-band center), M (d-band filling), and NM (p-band filling) derived from the density of states (DOS) were calculated using the Python *pymatgen* package.^71^

X-band filling (X = s, p, d) was calculated as
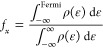
4Here, ε means energy and ρ(ε) means the density of states.

The unoccupied d-band center was calculated as
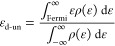
5

The doping formation energies were calculated to estimate the stability of the doped Mo_2_C systems under Mo_2_C synthesis conditions.^14,24^ Since Mo_2_C catalysts are synthesized by performing carburization with CH_4_ and H_2_,^30^ we considered the possibility of doping heteroatom as follows:
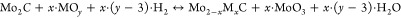
The formation Gibbs free energies (Δ*G*) of doped Mo_2-x_M_*x*_C were calculated as follows:
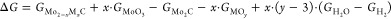
6Here, *G*_Mo_2-*x*_M_*x*_C_ is the Gibbs free energy of the slab upon doping with a hetero metal atom (M), *G*_Mo_2_C_ is the Gibbs free energy of the clean slab/pristine Mo_2_C, *G*_MO_*y*__ is the Gibbs free energy of the bulk of the most stable oxide phase of M at 600–700 °C, *G*_MoO_3__ is the Gibbs free energy of the bulk of the most stable oxide phase of Mo at 600–700 °C, *G*_H_2__ is the Gibbs free energy of H_2_, and *G*_H_2_O_ is the Gibbs free energy of H_2_O.

In this method, the vibrational and PV contributions of solids were neglected and the Gibbs free energies of Mo_2_C and Mo_2–*x*_M_*x*_C solids were approximated as their respective electronic energies computed by DFT, considering that the temperature has a negligible influence on the systematic energy. The chemical potential of the gas-phase compounds H_2_ and H_2_O depends on the experimental temperature (*T*) and their corresponding partial pressures (*p*). We assume that it complies with the ideal gas law and that the chemical potential of gas-phase H_2_ and H_2_O at the reference state of 0 K is μ_H_2__(0K) = *E*_H_2__ and μ_H_2_O_(0K) = *E*_*H*_*2*_O_, respectively. At arbitrary *T* and *p*, they can then be written as
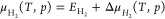
7
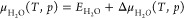
8

And thus, the formation Gibbs free energies (Δ*G*) can be written as
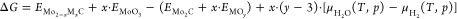
9

The MKMCXX package^72^ was used for the microkinetic modeling (MKM) simulations.^73^ MKM is a tool used to identify the critical intermediates and rate-determining elementary reactions. The chemokinetic network was modeled using a set of ordinary differential equations involving rate constants, surface coverages, and partial pressures of gas-phase species. Time integration of the differential equations was conducted using the linear multistep backward differential formula method with a relative and absolute tolerance of 10^–8^. The DFT results including the elementary reaction steps, forward/backward barriers, and forward/backward prefactors are provided in section S2 of the Supporting Information.

The rate constant (*k*) of an elementary reaction step is given as
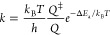
10where *Q*^⧧^ and *Q* are the partition functions of the activated complex and its corresponding initial state, respectively, and Δ*E*_a_ is the activation energy.

To identify the steps that control the butyric acid consumption rate and the product distribution, we performed the degree of rate control (DRC) analysis, as developed by Kozuch and Shaik^74,75^ and popularized by Campbell.^76^

The DRC coefficient is defined as
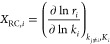
11

## 3 Results and Discussion

### 3.1 HDO Reaction Mechanism of Butyric Acid over β-Mo_2_C (101) Surface

The overall balanced equation for the HDO reaction mechanism is
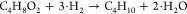


All elementary reaction steps are summarized in section 2 of the Supporting Information. In Tables S1 and S2, the reaction pathways and energetics for dissociation of H_2_ on the β-Mo_2_C (101) catalytic surface are summarized based on the DFT mechanism study. In this section the whole mechanism will be discussed as follows: (1) butyric acid to butanal; (2) butanal to butanol; (3) butanol to 2-butene; (4) 2-butene to butane.

#### 3.1.1 Butyric Acid to Butanal


**Figure 1:**
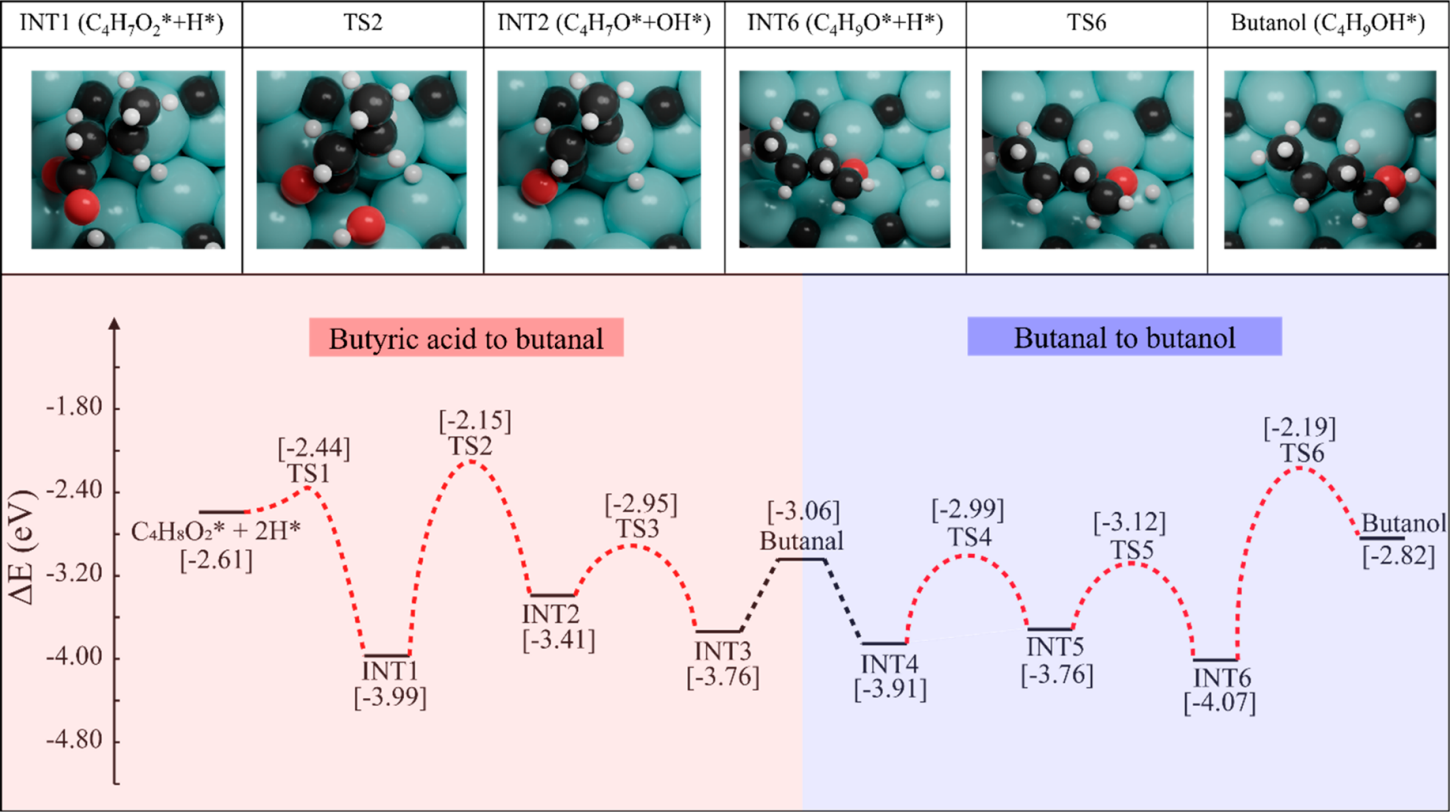
Reaction energy profile for the formation of butanal and butanol from butyric acid over the (101) surface of β-Mo_2_C. The local geometries of intermediates and transition states of the key reaction steps are highlighted on top.

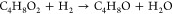
The reaction energy diagram is shown in Figure 1. As reported in the literature,^36^ butyric acid prefers to be adsorbed on the surface via metal (surface)–oxygen (reactant) interactions with an *E*_ads_ value of −0.80 eV. Upon reactant adsorption, the H from the C–OH bond tends to exothermically dissociate and stabilize on the surface, as shown in Figure 1, INT1. Subsequently, breaking of the C–O bond and forming the OH group on the surface take place simultaneously, which requires an activation energy (*E*_a_) of 1.84 eV, and this process (Figure 1, INT1 → INT2) is endothermic in nature (Δ*E* = 0.58 eV). The next step is hydrogenation of the C atom of the carbonyl group, which can be achieved with an activation barrier of 0.46 eV. The resulting product, butanal, is stable on the surface due to strong metal–carbon (reactant) and carbon (surface)–carbon (reactant) interactions, and its desorption from the surface is difficult (Δ*E* = 3.17 eV). The OH group on the surface can interact with H on the surface to form H_2_O (Tables S3 and S4).

#### 3.1.2 Butanal to Butanol


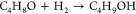
To convert butanal to butanol, the first step (Figure 1, INT4 → INT5) is the hydrogenation of the terminal C (*E*_a_ = 0.92 eV; Δ*E* = 0.15 eV) and subsequent hydrogenation (Figure 1 INT6 → INT7) of O (*E*_a_ = 1.88 eV; Δ*E* = 1.25 eV). The hydrogenation order cannot be the other way around, as the resulting intermediate is unstable. Also, hydrogenation of O on the surface is a kinetically demanding process (*E*_a_ = 1.41 eV, Δ*E* = 0.55 eV) as reported in Tables S3 and S4, which is consistent with a previous report^36^ in the literature. The barrier for butanol desorption from the surface is 3.00 eV.

#### 3.1.3 Butanol to 2-Butene


**Figure 2:**
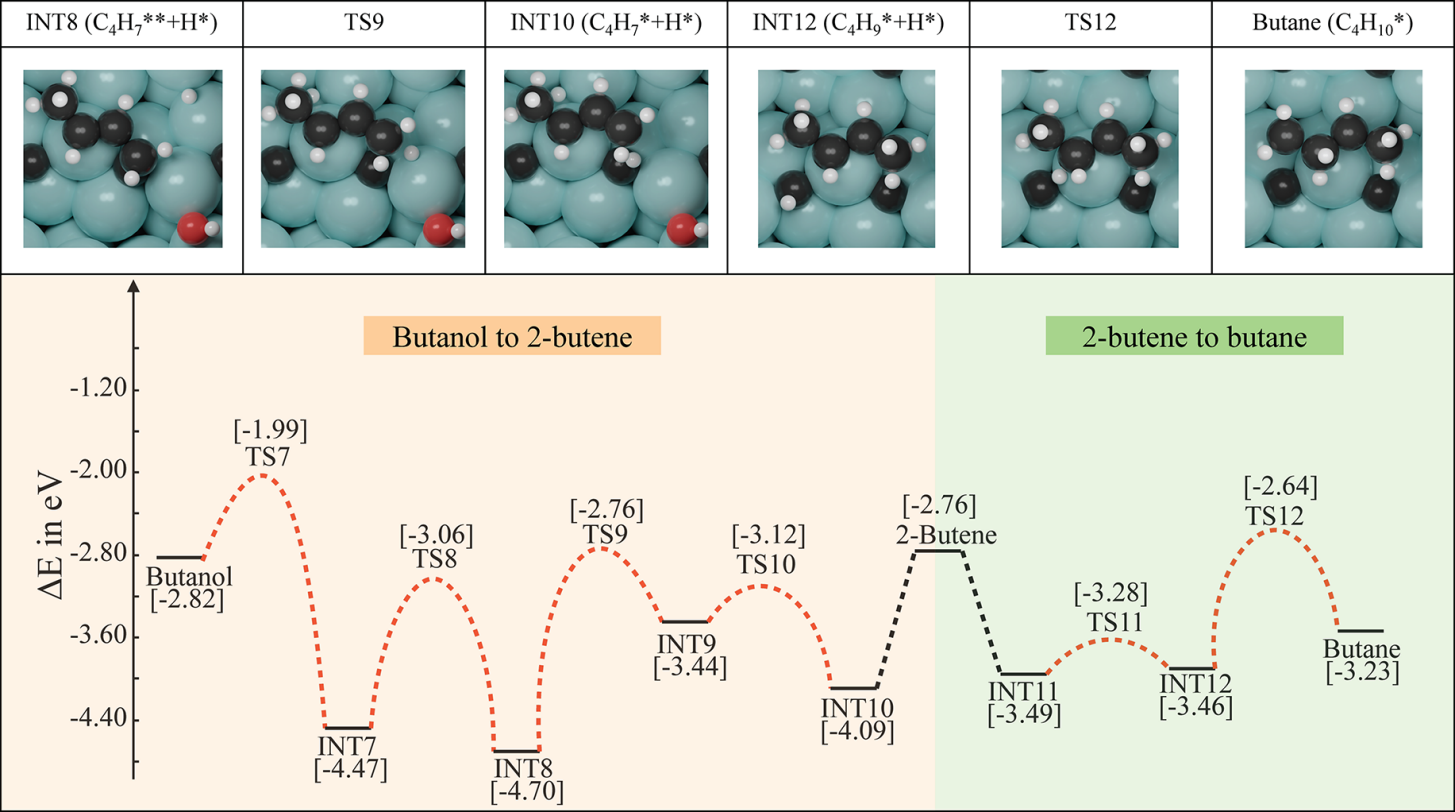
Reaction energy profile for the formation of 2-butene and butane from butanol over the (101) surface of β-Mo_2_C. The local geometries of intermediates and transition states of the key reaction steps are highlighted on top.

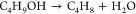
For the conversion of butanol to butene, pathways for both 1-butene and 2-butene formation were considered; however, we were unable to locate a transition state for the formation of 1-butene. The reaction energy diagram of butanol to 2-butene is shown in Figure 2. In the first step, activation of the C–OH bond in butanol takes place, which is kinetically and thermodynamically favorable (*E*_a_ = 0.83 eV; Δ*E* = −1.65 eV). Interestingly, the barrier for C–OH bond activation we obtained is significantly smaller than that reported by Shi et al.,^36^ i.e., 1.50 eV. This could be because that surface C atoms facilitate C–OH bond activation, while in ref (36) only Mo atoms are present on the surface. The obtained C_4_H_9_* (INT7) species is stable on the surface. In the next step, C_4_H_9_* undergoes dehydrogenation to produce a precursor, C_4_H_7_** (INT8), for 2-butene formation. This process involves H-transfer from C2 to the surface (*E*_a_ = 1.41 eV; Δ*E* = −0.13 eV), which also triggers H-transfer from C3 to the surface, as it was being stabilized by C3 from the reactant and Mo from the surface. This further stabilizes INT8 because of the strong C–C interaction between C_reactant_ and C_surface_. To push the reaction forward, the C1–C_surface_ bond cleavage was triggered by the diffusion of an H atom to C_surface_ (Figure 2, INT8 → INT9), which is kinetically difficult and thermodynamically endothermic (*E*_a_ = 1.94 eV; Δ*E* = 1.26 eV). The final step is hydrogenation of C1 to form 2-butene (Figure 2, INT9 → INT10). This step needs to overcome a barrier of 0.32 eV, and the reaction energy is −0.65 eV. The desorption barrier for 2-butene from the surface is 2.25 eV.

#### 3.1.4 2-Butene to Butane


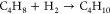
The final step in the HDO of butyric acid is the formation of butane. The C=C bond of 2-butene can be hydrogenated via a two-step mechanism. The 2-butene intermediate (INT11) is very stable on the surface (*E*_ads_ = −2.76 eV). To promote the hydrogenation reaction (INT11 → INT12), surface H* needs to be activated. First, a one-step dual hydrogenation was considered. However, the 2-butene species resists hydrogenation by one of the H atoms because of its high stability. Hence, the hydrogenation steps were carried out via a two-step mechanism. C2 was first hydrogenated (*E*_a_ = 0.21 eV; Δ*E* = 0.03 eV; INT11 → INT12) followed by C3 hydrogenation (*E*_a_ = 0.82 eV; Δ*E* = 0.23 eV; INT12 → butane).

### 3.2 Microkinetic Modeling Based on DFT Results

**Figure 3:**
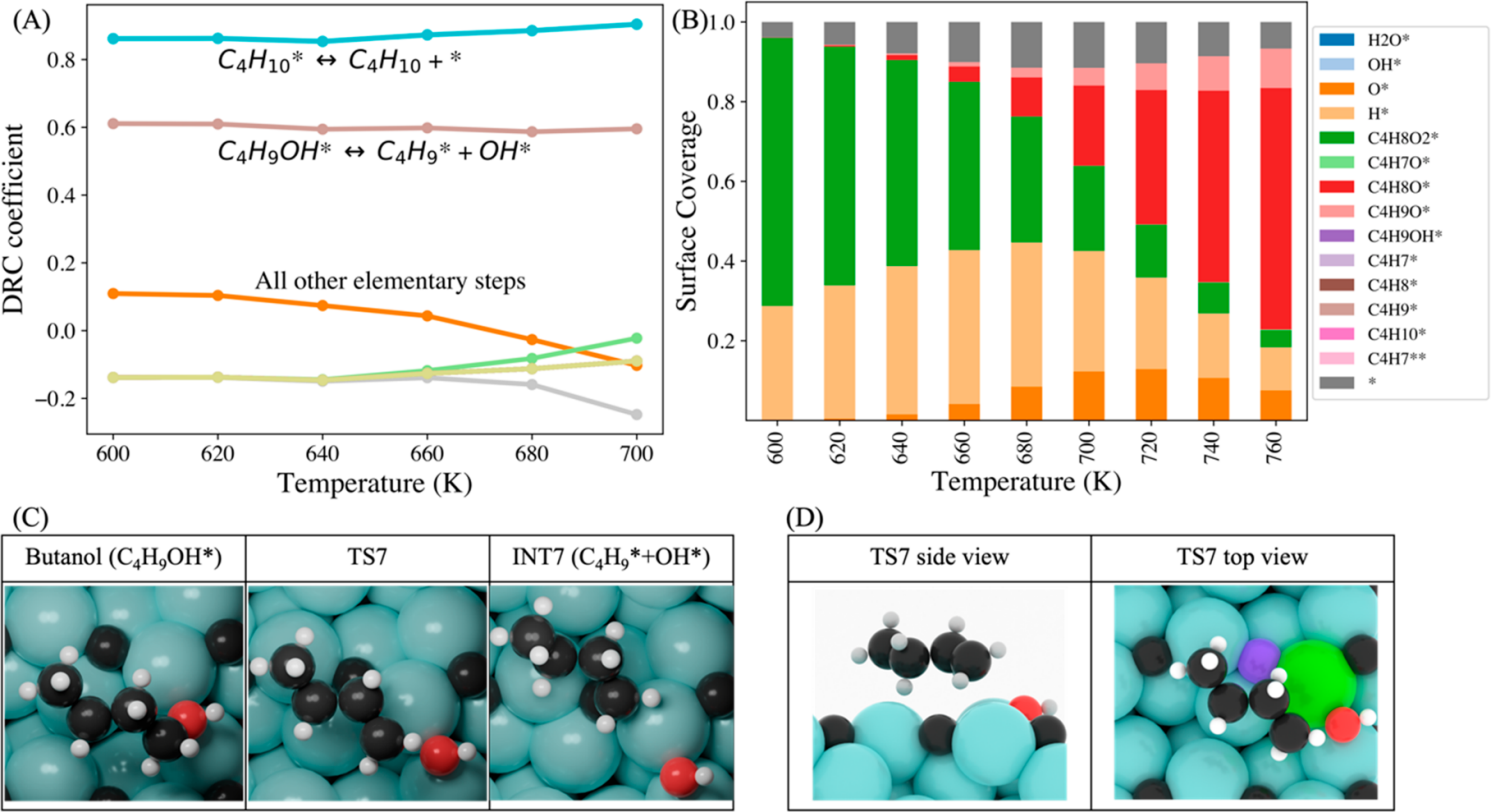
(A) DRC coefficients for all the elementary reaction steps as a function of temperature. (B) Surface coverage as a function of temperature. (C) Local geometries of the rate-determining step identified by DRC analysis. (D) Side and top views of the transition state of the RDS with active sites for heteroatom doping highlighted (green, Mo active site for metal doping; purple, C active site for nonmetal doping).

To further unravel the rate-determining step under experimental reaction conditions (*T* = 623 K, *p*_H_2__ = 30 bar),^24^ MKM was performed, and the results are presented in Figure 3A,B. From the DRC analysis (Figure 3A), we could see that the overall reaction rate is the most sensitive to butanol dissociation and butane desorption elementary steps. As DFT-D3 is known to overestimate the binding energies of the reactants on the solid surface,^64,77^ the butanol dissociation elementary step is assigned to be the RDS, and the local geometries are highlighted in Figure 3C. It is important to highlight here that butanol dissociation was reported to be the RDS in the work of Shi et al.^36^ as well, in which they studied the HDO reaction of butyric acid over a hexagonal Mo_2_C (101) surface. From our DFT and MKM results, it is found that the (101) facet of the orthorhombic Mo_2_C with mixed surface termination is more active than the (101) facet of the hexagonal Mo_2_C with only Mo sites on the surface. The activation barrier for breaking the C–OH bond of butanol is 0.83 eV in our case, much lower than that over the hexagonal Mo_2_C (101) surface (*E*_a_ = 1.50 eV).^36^ Moreover, the surface coverage analysis (Figure 3B) as a function of *T* reveals that as the reaction moves forward, the coverage of oxygen on the surface is increasing, which hints toward the plausible reaction route to molybdenum oxycarbide formation during the reaction. This is consistent with the observation that water formation on the β-Mo_2_C (101) surface is kinetically (*E*_a_ = 1.37 eV) and thermodynamically (Δ*E* = 1.00 eV) unfavorable (Tables S3 and S4). Instead, the OH groups produced on the surface during the reaction can further dissociate to produce an O species on the surface, which could eventually lead to the formation of molybdenum oxycarbide (*E*_a_ = 0.86 eV; Δ*E* = −0.55 eV). Also, reaction orders of the reactants, i.e., butyric acid (C_4_H_8_O_2_) and gas-phase hydrogen (H_2_), are calculated as a function of *T* (section S8 in the Supporting Information). Figure S5 shows that at higher temperatures the hydrogen is easily accessible for the reaction; hence, increasing the concentration of H_2_ in the reaction could potentially speed up the reaction, while the reaction order of butyric acid is not very sensitive to the temperature. Selectivity analysis is shown in Figure S6. It is demonstrated that for all four products, i.e., butanal, butanol, 2-butene, and butane, butene is the main product over the surface of β-Mo_2_C (101) throughout the simulated reaction temperatures.

### 2.3 Heteroatom Doping and Descriptor–Activity Relationship

**Figure 4:**
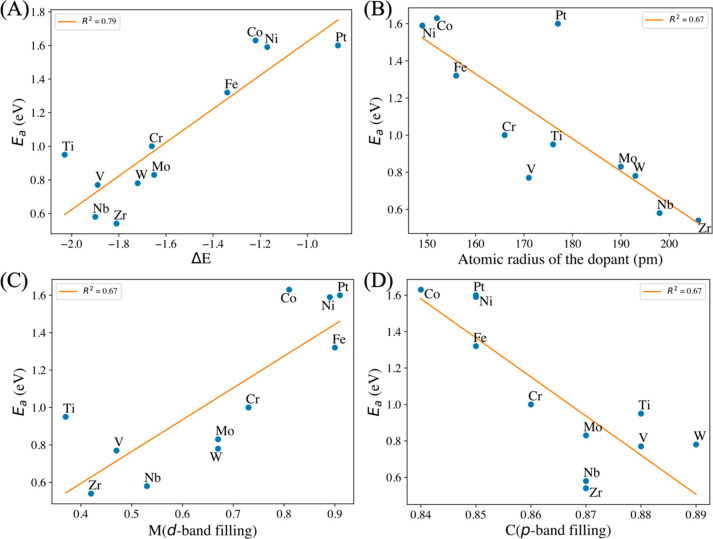
Linear-scaling relationships of descriptors: (A) reaction energy, Δ*E*; (B) *atomic radius* of the dopant; (C) d-band filling of the dopant; (D) p-band filling of surface C with the reaction barriers of the RDS.

Based on the MKM result, i.e., butanol dissociation (C_4_H_9_*OH + * → C_4_H_9_* + *OH) is the RDS in HDO of butyric acid to butane (see above and Figure 3A), we then explored ways to improve the activity of Mo_2_C for butanol dissociation. From the transition state calculation, it is evident that the kinetic barrier of butanol dissociation mostly depends on how well the Mo active site (Figure 3D, green) can stabilize the OH group and how well the C active site (Figure 3D, purple) can stabilize the C_4_H_9_* group. Hence, we modified the property of the metal (M) and nonmetal (NM) actives sites in the pristine Mo_2_C surface by heteroatom doping, as previously demonstrated by Wang et al. and Jiang et al.^39,53^ In this study, the doped catalysts are representative of a single-atom catalyst model with a low loading of dopants. It is therefore assumed that the heteroatom dopant sites are highly dispersed in the matrix of Mo_2_C, and dopants do not change the main crystal structure of Mo_2_C. The M active site was doped by a series of transition metals (M = Ti, V, Cr, Fe, Co, Ni, Pt, Nb, Zr, W) where the OH group adsorbs, and the NM active sites by other main-group elements (NM = B, N, O, S) were examined. The doping of the NM active site did not change the barrier for RDS significantly (Figure S4), but doping of the M active site resulted in a wide range of barriers for the RDS (Figure 4A). The barrier of RDS on the pristine Mo_2_C surface is 0.83 eV, as discussed in section 3.1. Upon doping with Zr, the barrier of RDS can be reduced to 0.54 eV, while, upon doping with Co, it was increased to 1.63 eV. It was found that doping with Zr, Nb, V, and W enhances the activity of the Mo_2_C surface, whereas doping with Ti, Cr, Fe, Co, Ni, and Pt inhibits the activity of the Mo_2_C surface. To further extract the intrinsic properties of dopants governing the activity, electronic properties such as the Fermi level (*E*_F_), Bader charge (M), Bader charge (NM), M (d-band center), NM (p-band center), M (d-band filling), and NM (p-band filling) of all the doped surfaces were studied, and the relationships between the electronic and geometric descriptors and the activation barriers for RDS (C–OH cleavage) were interrogated (Figure S3). With such a study, we aimed to establish structure–activity relationships by correlating the physical and/or chemical descriptors with the C–OH bond cleavage barrier. It was found that the reaction follows the BEP principle; i.e., the activation energy is inversely proportional to the reaction enthalpy (Figure 4A). Interestingly, the intrinsic geometric descriptor of the *atomic radius* of dopants has a good correlation with the activation barrier of C–OH bond cleavage (Figure 4B). Metal dopants with larger radii exhibit higher activity for C–OH bond breaking, suggesting that a flexible coordination environment plays a key role in tuning the barrier of the RDS. The other two relevant descriptors identified are M (d-band filling) and NM (p-band filling), as shown in Figure 4C,D. Furthermore, we did a multilinear regression^78^ using the Python *scikit-learn*(79) package for the hitherto mentioned most-promising descriptors and all other electronic and geometric data (Figure S3). It was shown that Δ*E*, M (d-band filling), NM (*p*-band filling), and the radius of M have good linear-scaling relationships with the activation barriers (Figure 4 and Figure S3).

**Figure 5:**
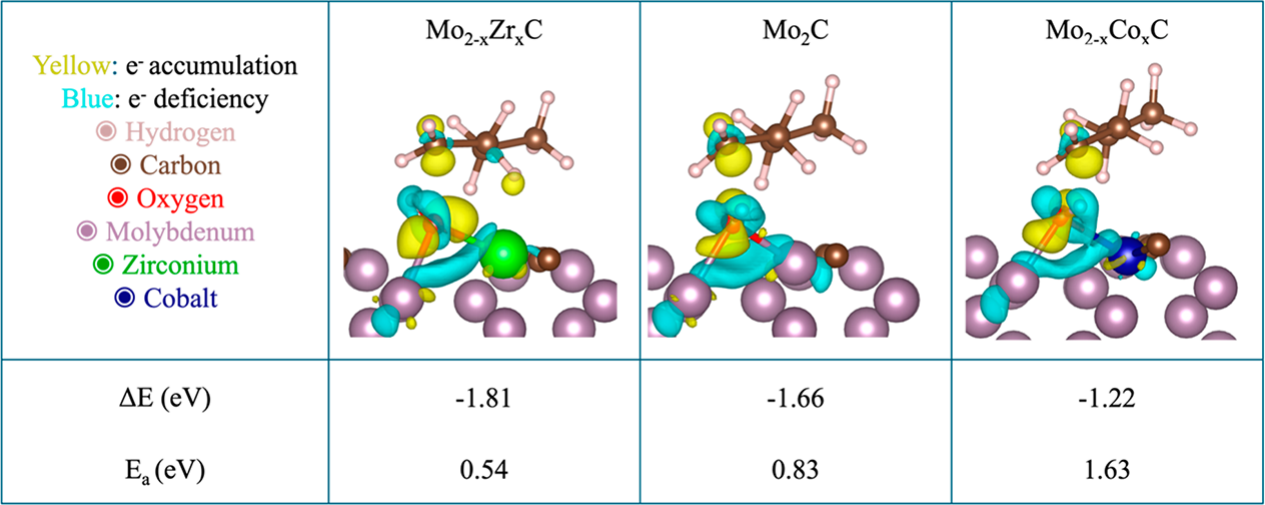
Charge density difference analysis of the proposed Mo_2–*x*_Zr_*x*_C (best), pristine Mo_2_C, and Mo_2–*x*_Co_*x*_C (worst) catalysts. Isosurface value: 0.0074.

Here, M (d-band filling) is the fractional occupation of the d-band of the doped active M site, while NM (p-band filling) is the fractional occupation of the p-band of the doped active NM site. Figure 4C shows that it is relatively easier to break the C–OH bond if the d-band of the doped M site is emptier compared to the d-band of the Mo active site in the pristine Mo_2_C catalyst. On the other hand, Figure 4D shows that it is relatively easier to break the C–OH bond if the p-band of the NM site is fuller compared to the p-band of the C active site adjacent to the Mo active site in the pristine Mo_2_C catalyst. It can be inferred that electrons are being transferred from the M (d-band) → C (p-band). This is similar to an observation by Wang et al., where they observe strong electron transfer from the dopant metal to the surface.^39^ This charge transfer makes the M (d-band) more accessible to stabilize the −OH group by accepting electrons from the O (p-band). To obtain a physical picture of this charge transfer process, a charge density difference analysis was performed (Figure 5), as described in section S6 in the Supporting Information. It can be seen that electron transfer from the O (p-band) → M (d-band) is the most significant for the Mo_2–*x*_Zr_*x*_C catalyst because the d-band of Zr is emptier, and hence the activation barrier is lower. However, in the case of Mo_2-x_Co_*x*_C, the d-band of Co is relatively full (Figure 4C); therefore, O (p-band) → M (d-band) electron transfer is not favorable, which is reflected by a larger *E*_a_ compared to that of the pristine Mo_2_C catalyst. It is worth mentioning that improving the barrier of the RDS by means of heteroatom doping could cause an imbalance in the overall reaction and shift the RDS to another elementary step. Therefore, further calculations would be needed to verify if the RDS is shifted.

### 2.4 Formation Gibbs Free Energy of Doped Mo_2–*x*_M_*x*_C

**Figure 6:**
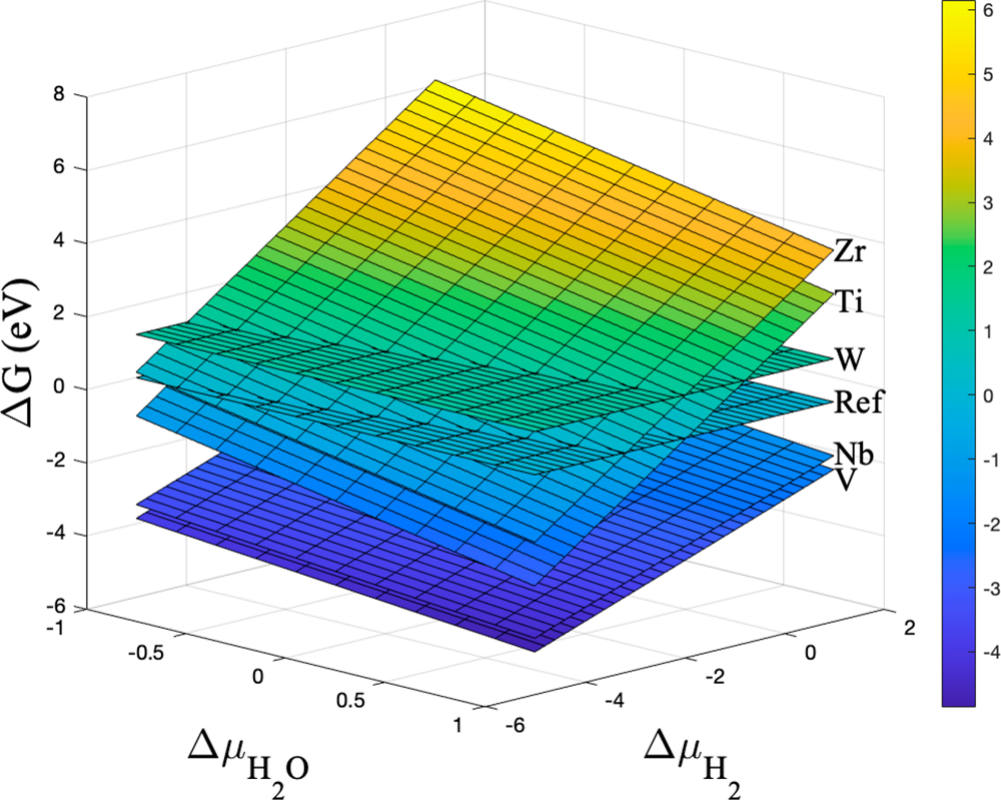
Formation Gibbs free energy of doped Mo_2_C as a function of the H_2_ and H_2_O chemical potentials.

After we identified the most-promising dopants (M = Zr, Nb, V, Ti, W) for enhancing the activity of pristine Mo_2_C, we further calculated the formation Gibbs free energy (Δ*G*) as described in section 2, eqs 6–9, and Jiang et al.’s work^53^ to evaluate the thermodynamic facility of forming these doped Mo_2_C under experimental Mo_2_C synthesis conditions.^14,24^Figure 6 demonstrates the formation Gibbs free energy as a function of the chemical potentials of H_2_ and H_2_O. It is indicated that Nb- and V-doped Mo_2_C are the most facile doped catalysts to synthesize, while the synthesis of Ti- and Zr-doped Mo_2_C is the least feasible under the typical experimental carburization conditions. Contrastingly, synthesis of W-doped Mo_2_C is independent of the chemical concentration of H_2_ and H_2_O. From these results it is speculated that even though DFT predicts the lowest barrier for RDS (0.54 eV) in the case of Mo_2–*x*_Zr_*x*_C, its synthesis under typical Mo_2_C synthesis conditions could be quite challenging. On the other hand, Mo_2–*x*_Nb_*x*_C is not only relatively much easier to form compared to Mo_2–*x*_Zr_*x*_C, but also can enhance the activity of the Mo_2_C catalytic surface as well (barrier for RDS: 0.58 eV) and hence is a good candidate for experimental validation. Overall, it is concluded that Mo_2–*x*_Zr_*x*_C and Mo_2–*x*_Nb_*x*_C are the two most-promising catalysts for enhancing the activity of Mo_2_C for HDO reactions.

## 4 Conclusions

In this work, the comprehensive HDO reaction mechanism of butyric acid over the β-Mo_2_C (101) catalytic surface has been studied by means of DFT. Combining the DFT results with MKM, it is identified that C–OH activation during the butanol dissociation is the rate-determining step for the HDO of butyric acid. It is found that water dissociation on the surface of Mo_2_C is favorable, which could contribute to molybdenum oxycarbide formation during the reaction. The insights into the rate-determining step were then exploited by fine-tuning the activity of Mo_2_C by means of heteroatom doping. The results indicate that Mo_2_C catalytic surfaces upon doping with Zr and Nb have significantly lower activation barriers toward C–OH bond dissociation compared to the pristine Mo_2_C surface. Moreover, the doping data was further analyzed using multilinear regression to unravel the most important descriptors, and it is established that generic geometric (dopants’ *atomic radius*) and electronic (dopants’ d-band filling) descriptors play a key role in governing the activity of the doped Mo_2_C catalysts.

## Supporting Information Available

Atomic models used for DFT calculations, elementary reaction steps with forward and backward barriers (and prefactors) used for MKM simulations, calculations for H_2_ dissociative adsorption and H_2_O formation/dissociation, charge density difference analysis, correlation heat map for potential descriptors of reactivity, and the rest of the MKM results (PDF)
