# Supplementary material for: Toward the Rational Design of More Efficient Mo2C Catalysts for Hydrodeoxygenation–Mechanism and Descriptor Identification
Source: ACS Catal. 2023 Oct 5;13(20):13446–55. doi: 10.1021/acscatal.3c03728 (PMC10594588; doi:10.1021/acscatal.3c03728)
Supplement: Supplementary file 1 — cs3c03728_si_001.pdf [file cs3c03728_si_001.pdf]

Supporting Information

Towards rational design of more efficient Mo_2C catalysts for hydrodeoxygenation – mechanism and descriptor identification

Raghavendra Meena,[†,‡] Johannes Hendrik Bitter,[†] Han Zuilhof,[‡,∮] Guanna Li[*,†,‡]

†Biobased Chemistry and Technology, Wageningen University, Bornse Weilanden 9,

6708 WG Wageningen, The Netherlands

‡Laboratory of Organic Chemistry, Wageningen University, Stippeneng 4, 6708 WE

Wageningen, The Netherlands

∮School of Pharmaceutical Sciences and Technology, Tianjin University, 92 Weijin Road, Tianjin,

300072, China.

E-mail: guanna.li@wur.nl

S1. Surface slab model

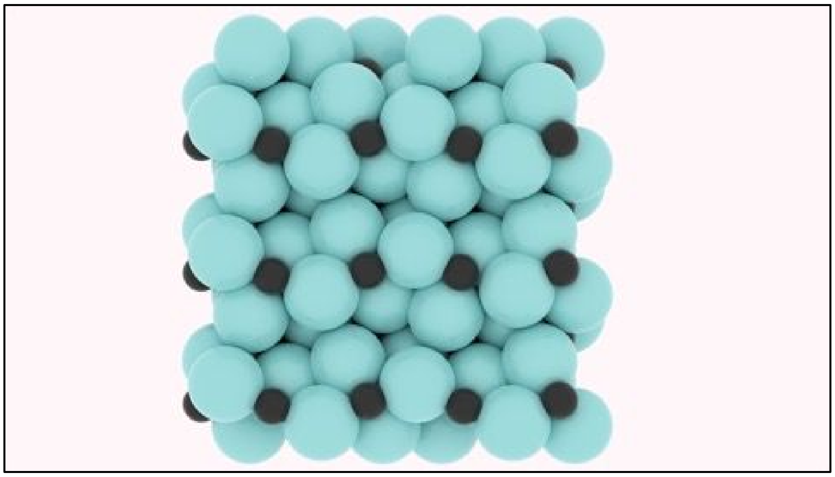

Figure S1: β-Mo_2C (101) surface, top view. Color key: Molybdenum (Mo), Carbon (C), Oxygen (O), Hydrogen (H).

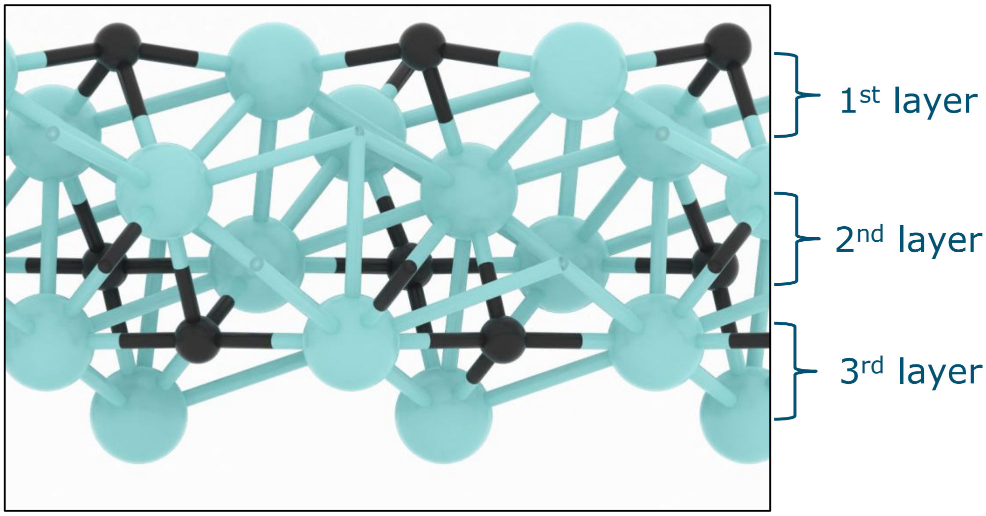

Figure S2: β-Mo_2C (101) surface, side view highlighting three stoichiometric layers of Mo_2C. Color key: Molybdenum (Mo), Carbon (C), Oxygen (O), Hydrogen (H).

S2. Elementary steps & input for microkinetic modelling

The hydrodeoxygenation reaction of butyric acid to butane was explored, and the whole mechanism was divided in four sections as following:

1. butyric acid to butanal:

   1.1. H_2 + 2*←→ 2 H*

   1.2. $C_4H_8O_2$ + * ←→ $C_4H_8O_2$*

   1.3. $C_4H_8O_2$* + * ←→ $C_4H_7O_2$* + H*

   1.4. $C_4H_7O_2$* + H* ←→ C_4H_7O* + OH* [INT1 → INT2]

   1.5. C_4H_7O* + H* ←→ C_4H_8O* + * [INT2 → INT3]

   1.6. C_4H_8O* ←→ C_4H_8O + *

   1.7. OH* + H* ←→ H_2O*

   1.8. H_2O* ←→ H_2O

2. butanal to butanol:

   2.1. H_2 + 2*←→ 2 H* [Butanal → INT4]

   2.2. C_4H_8O* + H* ←→ C_4H_9O* + * [INT4 → INT5]

   2.3. C_4H_9O* + H* ←→ C_4H_9O* + H* [H-diffusion from C site to Mo-Mo bridge site, INT6]

   2.4. C_4H_9O* + H* ←→ C_4H_9OH* + * [INT6 → Butanol]

2.5. $C_4H_9OH^*$ ←→ C_4H_9OH + *

3. butanol to 2-butene:

3.1. $C_4H_9OH^*$ + * ←→ $C_4H_9^*$ + OH^* [Butanol → INT7]

3.2. $C_4H_9^*$ + 3* ←→ $C_4H_7^{**}$ + 2H* [INT7 → INT8]

3.3. $C_4H_7^{**}$ + H* ←→ $C_4H_7^*$ + H* + * [INT8 → INT9]

3.4. $C_4H_7^*$ + H* ←→ $C_4H_8^*$ + * [INT9 → INT10]

3.5. $C_4H_8^*$ ←→ C_4H_8 + *

4. 2-butene to butane:

4.1. H_2 + 2*←→ 2 H* [2-butene → INT11]

4.2. $C_4H_8^*$ + H* ←→ $C_4H_9^*$ + * [INT11 → INT12]

4.3. $C_4H_9^*$ + H* ←→ $C_4H_{10}^*$ + * [INT12 → Butane]

4.4. $C_4H_{10}^*$ ←→ C_4H_{10} + *

4.5. OH* + H* ←→ H_2O^*

4.6. H_2O^* ←→ H_2O

The input data including the elementary reaction steps, forward/backward barriers ($E_{f,}$ E_b), forward/backward pre-factors ($A_{f,}$ A_b) is provided below.

Elementary reactions	A_f	A_b	E_f (eV)	E_b (eV)
H_2 + 2*←→ 2 H*	1,00E+09	1,00E+15	0,05	0,80
$C_4H_8O_2$ + * ←→ $C_4H_8O_2$*	1,00E+09	1,00E+15	0,00	0,81
$C_4H_8O_2$* + H* ←→ C_4H_7O* + OH*	1,00E+13	1,00E+13	1,83	1,26
C_4H_7O* + H* ←→ C_4H_8O* + *	1,00E+13	1,00E+13	0,47	0,82
C_4H_8O* ←→ C_4H_8O + *	1,00E+15	1,00E+09	3,15	0,00
OH* + H* ←→ H_2O*	1,00E+13	1,00E+13	1,37	0,38
OH* + * ←→ O*+ H*	1,00E+13	1,00E+13	0,86	1,41
H_2O* ←→ H_2O	1,00E+15	1,00E+09	0,44	0,00
C_4H_8O* + H* ←→ C_4H_9O* + *	1,00E+13	1,00E+13	0,92	0,77
C_4H_9O* + H* ←→ C_4H_9OH* + *	1,00E+13	1,00E+13	1,88	0,62
C_4H_9OH* ←→ C_4H_9OH + *	1,00E+15	1,00E+09	2,99	0,00
C_4H_9OH* + * ←→ C_4H_9* + OH*	1,00E+13	1,00E+13	0,82	2,47
C_4H_9* + 3* ←→ C_4H_7** + 2H*	1,00E+13	1,00E+13	1,41	1,64
C_4H_7** + H* ←→ C_4H_7* + H*	1,00E+13	1,00E+13	1,94	0,68
C_4H_7* + H* ←→ C_4H_8* + *	1,00E+13	1,00E+13	0,32	0,97
C_4H_8* ←→ C_4H_8 + *	1,00E+15	1,00E+09	2,25	0,00
C_4H_8* + H* ←→ C_4H_9* + *	1,00E+13	1,00E+13	0,20	0,17
C_4H_9* + H* ←→ C_4H_{10}* + *	1,00E+13	1,00E+13	0,82	0,59
C_4H_{10}* ←→ C_4H_{10} + *	1,00E+15	1,00E+09	2,30	0,00

S3. H_2 dissociation on β-Mo_2C (101) catalytic surface

Three unique adsorption sites for the physisorption of H_2 were identified as shown in Tab. S1. The best possible scenario is Tab. S1 configuration (#1). In which the H_2 is weakly adsorbed over the exposed layer of Mo (top layer). The activation barrier (E_a) for physisorption of H_2 in this case is almost a barrier-less process (E_a = 0.05eV). Upon physisorption, H_2 can further dissociate in three unique ways as shown in Tab. S2. Upon relaxation over all the possible adsorption sites in Tab. S2, configuration (#1) was established as the preferred location for the adsorption of H atoms with an adsorption energy (E_{ads}) of -0.92 eV. This configuration could be obtained by overcoming a barrier of 0.77eV.

Table S1: Physisorption of H_2 molecule on three unique active sites. Color key: Molybdenum (Mo), Carbon (C), Hydrogen (H).

Configuration	Top view	Side view	E_{ads} (eV)
(#1) H_2 on Mo (top layer)	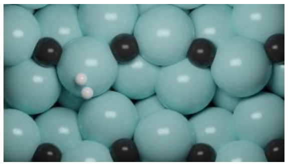	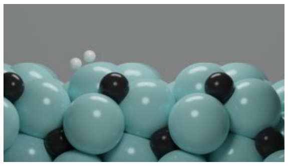	-0.12
(#2) H_2 on Mo (second layer)	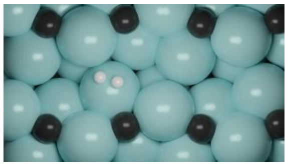	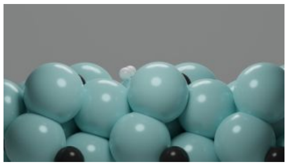	0.13
(#3) H_2 on C (top layer)	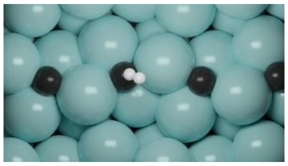	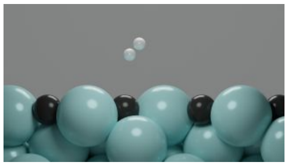	0.41

Table S2: Unique pathways for dissociation of H_2 molecule on the Mo_2C catalytic surface. Color key: Molybdenum (Mo), Carbon (C), Hydrogen (H).

Configuration	Top view	Side view	E_{ads} (eV)
(#1) Heterogenous cleavage over C (top layer) and Mo’s in the first-two layers	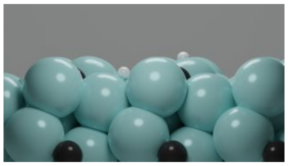	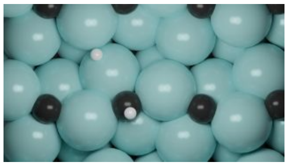	-0.92
(#2) Homogeneous cleavage over C’s in the top layer	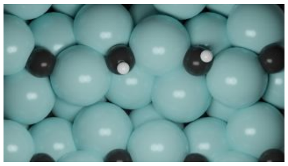	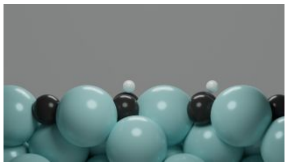	-0.75
(#3) Homogeneous cleavage over Mo’s in the first-two layers	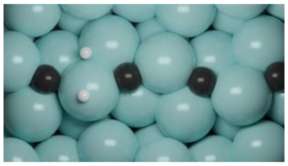	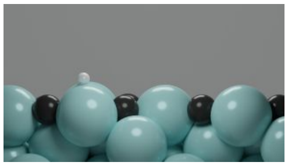	0.21

S4. Water formation and OH group dissociation over β-Mo_2C (101) surface

$$H_2O^* \Leftrightarrow OH^* + H^* \Leftrightarrow O^* + 2H^*$$

During the HDO reaction, C-O bonds are broken, and as a result, O (or OH) species were produced on the surface. The OH species can either form water by combining with an H species on the surface or further dissociate into O and H species. Both reaction pathways were studied, and it was found that water formation (Tab. S3) from OH and H on the surface is kinetically and thermodynamically a difficult process ($E_a = 1.37$ eV; $\Delta E = 1.00$ eV). In contrast, OH dissociation (Tab. S4) into O and H is relatively much easier compared to H_2O formation ($E_a = 0.86$ eV; $\Delta E = -0.55$ eV).

Table S3: Water formation on Mo_2C (101) surface. Color key: Molybdenum (Mo), Carbon (C), Oxygen (O), Hydrogen (H).

OH* + H*	TS	H_2O^*	Energetics
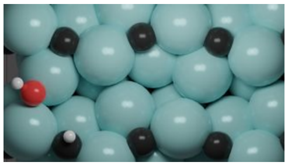	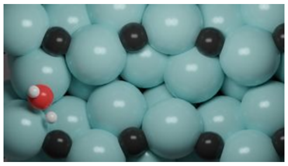	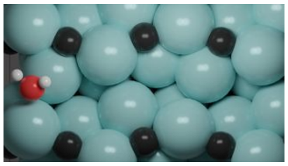	$E_a = 1.37$ eV,<br>$\Delta E = 1.00$ eV

Table S4: OH dissociation on Mo_2C (101) surface. Color key: Molybdenum (Mo), Carbon (C), Oxygen (O), Hydrogen (H).

OH*	TS	O* + H*	Energetics
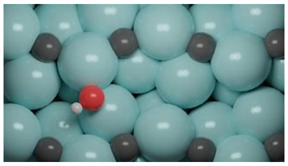	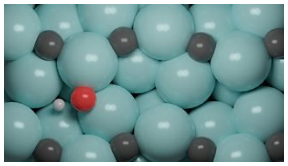	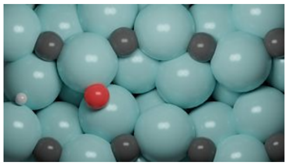	$E_a = 0.86$ eV,<br>$\Delta E = -0.55$ eV

S5. Descriptor identification

To identify the best descriptors of reactivity of Mo_2C catalytic surface, Pearson's correlation of each possible electronical and geometrical descriptor was calculated. The heatmap given in Fig. S3 was then used to identify the best descriptors of reactivity. The analysis was performed using the Python *pymatgen* package. The proposed active M and NM sites are highlighted in Fig. 3C of the main text.

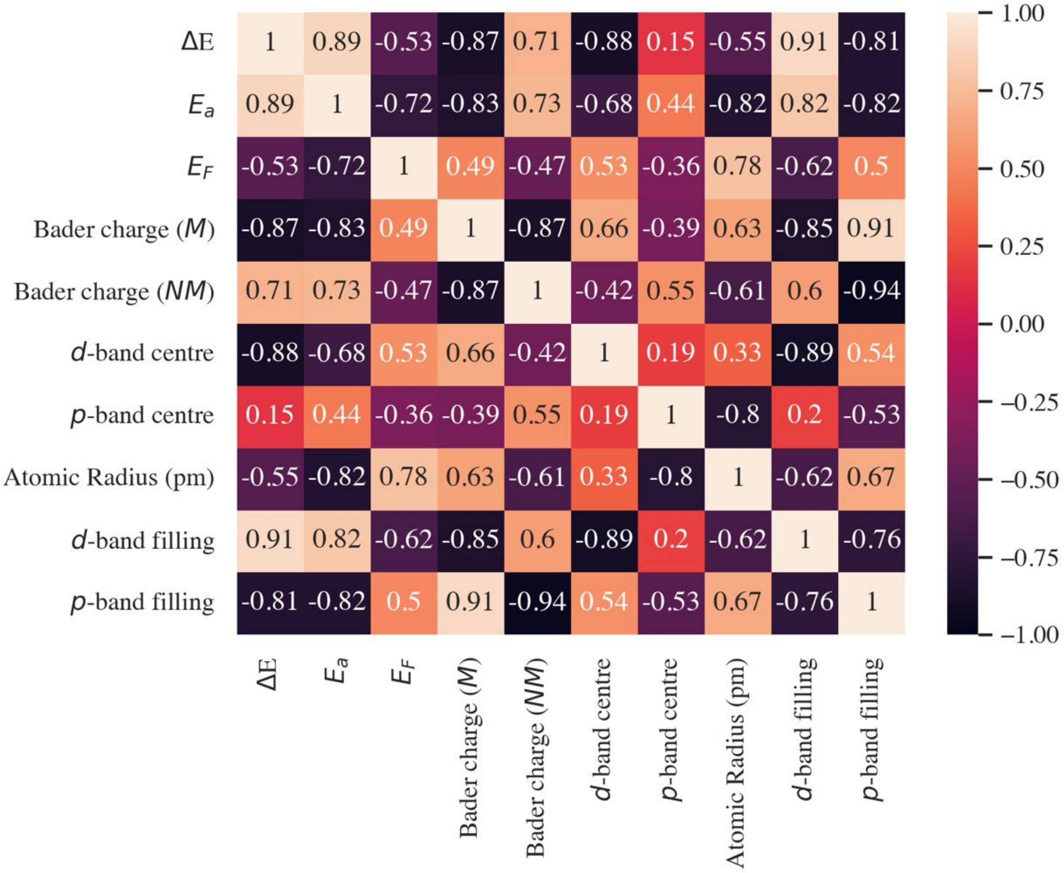

Figure S3: Pearson's correlation table for all the possible electronical and geometrical descriptors with the activation barrier of rate-determining step of C-OH breaking.

In the Figure, the following key is used for naming:

1. ΔE: reaction energy of the rate determining elementary step,

2. E_a: activation energy of the rate determining elementary step,

3. E_F: Fermi energy,

4. Bader charge (*M*): net Bader charge on the M,

5. Bader charge (*NM*): net Bader charge on the NM,

6. *d*-band centre: *d*-band centre of the M site,

7. *p*-band centre: *p*-band centre of the NM site,

8. *d*-band filling: *d*-band filling of the M site,

9. *p*-band filling: *p*-band filling of the NM site,

10. Atomic radius (pm): Atomic radius of the dopant.

S6. Charge density difference analysis using VESTA

The charge density differences ($\Delta\rho$) were calculated as follows:

$$\Delta\rho = \rho(Mo_2C + reactant) - \rho(Mo_2C) - \rho(reactant) \qquad \textit{Equation S1}$$

Here, $\rho(Mo_2C + reactant)$ is the charge density of the Mo_2C including the adsorbed reactant, $\rho(Mo_2C)$ is the charge density of the clean Mo_2C surface, and $\rho(reactant)$ is the charge density of the reactant in gas phase.

S7. Heteroatom doping of the active non-metal site

In efforts to enhance the activity of the Mo_2C catalyst, we also doped the non-metal active site as it can influence the nature of active metal site, as highlighted in Fig. 4C. We see that doping the non-metal active does not enhance the activity of Mo_2C catalyst. The differences in values of barrier of the RDS were too small to pursue non-metal active site doping for descriptor identification.

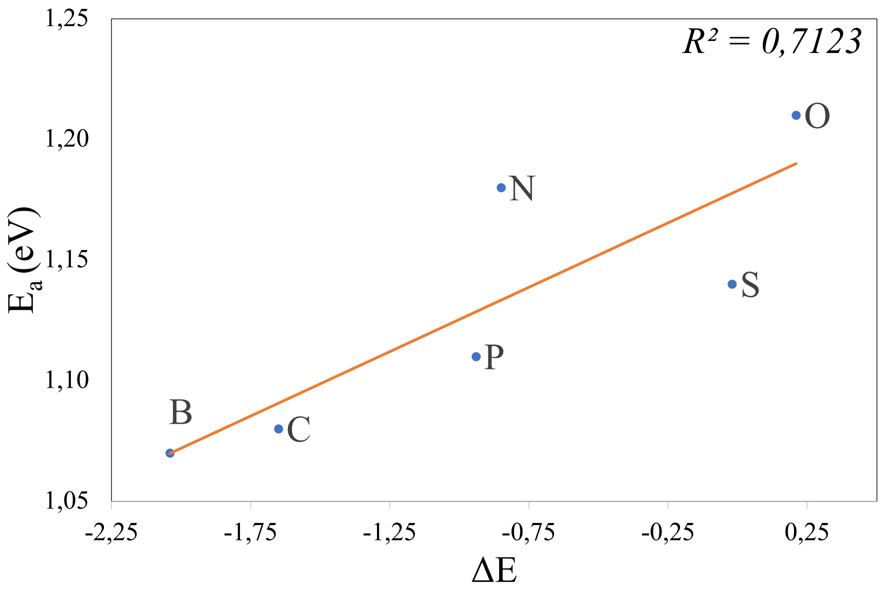

Figure S4: Linear scaling relationship between the barrier of RDS and reaction energy as function of heteroatom non-metal active site doping.

S8. Reaction order from microkinetic modelling

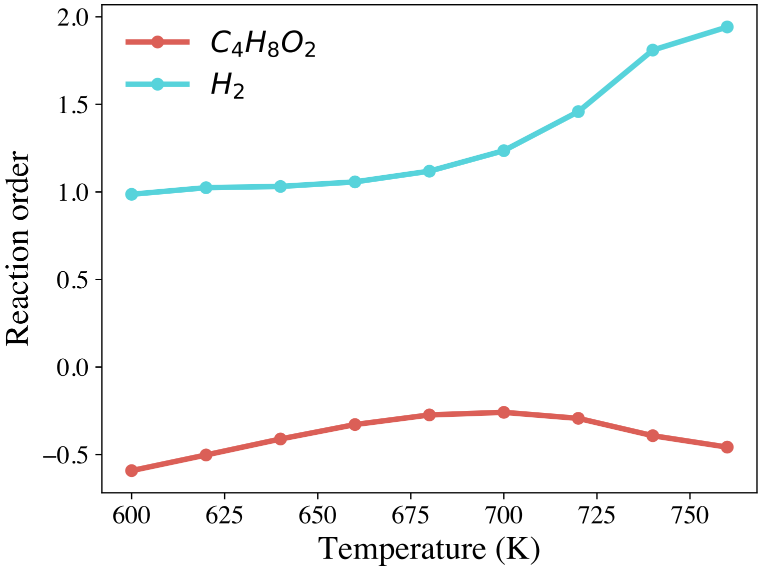

Figure S5: Reaction order of the reactants as a function of temperature.

S9. Selectivity analysis of different products using microkinetic modelling

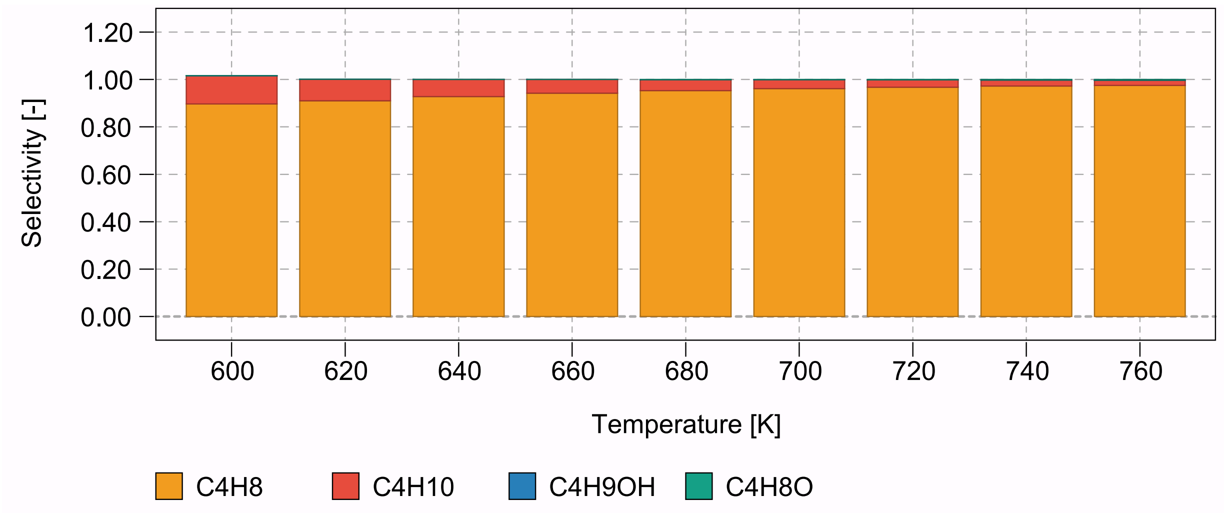

Figure S6: Selectivity analysis of different products as a function of temperature.
